# The Effects of Living Environment, Health Status of Family Members, and Migrant Elderly following Children’s Attitude about Care on Grandchildren’s Health Status in Weifang, China

**DOI:** 10.3390/children9091333

**Published:** 2022-09-01

**Authors:** Mingli Pang, Jieru Wang, Xiaoxu Jiang, Hexian Li, Shixue Li, Fanlei Kong

**Affiliations:** 1 Centre for Health Management and Policy Research, School of Public Health, Cheeloo College of Medicine, Shandong University, Jinan 250012, China; 2 NHC Key Lab of Health Economics and Policy Research, Shandong University, Jinan 250012, China

**Keywords:** living environment, attitude about care, family members, health status, migrant elderly following children

## Abstract

As urbanization is growing quickly in China, many migrant elderly following children (MEFC) migrate to big cities to care for their grandchildren (grandchildren of MEFC=GMEFC). This study aimed to explore the effects of the living environment, health statuses of family members, and MEFC’s attitude regarding the care of their children (children of MEFC=CMEFC) for their GMEFC on GMEFC’s health statuses in Weifang, China. Multistage cluster random sampling was used to select the participants, and 613 MEFC were included in total. Descriptive analysis, univariate analysis and binary logistic regression were used to investigate the association between the related variables and GMEFC’s health statuses. It was found that 74.9% of the GMEFC had excellent health statuses. The GMEFC who had siblings, the CMEFC with excellent health statuses, and the MEFC with excellent health statuses were more likely to have excellent health statuses. Moreover, the GMEFC who were female, elevators occasionally malfunctioned, the MEFC who were dissatisfied with the CMEFC’s time spent on caring, and the MEFC who did not understand or forgive the CMEFC’s limited time on caring were less likely to have GMEFC with excellent health statuses. The results indicated that a better living environment, better health statuses of family members, and a positive attitude of the MEFC regarding the care of CMEFC for GMEFC would result in a better health status of GMEFC.

## 1. Introduction

Children are a vulnerable group whose health not only affects their own lifelong well-being but also influences the future development of their nation, which necessitates more attention on children’s health statuses. Children’s health could be affected by genetic factors, as well as the quality of care from their caregivers [1]. Most elders in the family take part in baby-sitting in Chinese cities [2] and nearly half of the migrant elderly migrated from their hometown to another city in China to take care of their grandchildren [3]. Meanwhile, in the United States, a quarter of preschool children are regularly cared for by their grandparents [4]. As grandparents are a key source of caregiving for grandchildren, it is important to explore the grandparents’ influence on the health statuses of their grandchildren. In this study, the elderly who migrated following their children to take care of their grandchildren were defined as the MEFC [5], while the children of MEFC were designated as CMEFC and the grandchildren of MEFC were designated as GMEFC.

Previous studies have illustrated that living environment is strongly associated with health [6,7]. In the background of urban growth, aging populations and climate change, housing has become an increasingly important factor for health [8], and housing quality was significantly associated with mental health during the COVID-19 pandemic [9]. A study showed that early environmental pollution exposure in children under five years old was significantly related to anemia in sub-Saharan Africa [10]. Another study showed that indoor air pollution affected babies’ cognitive development [11]. Furthermore, a study in China showed that the cleanliness of the household environment had a positive effect on children’s health [12]. Moreover, the provision of housing, tenants’ experience of property quality and aspects of neighborhoods were significantly correlated with health [13].

The MEFC’s attitude about the caregiving provided by elders for the younger generation had been proven to be an important factor that influenced the health statuses of grandchildren [14,15]. Research had shown that the family atmosphere had a significant impact on children’s social and emotional development; that is, the better of the family atmosphere, the better the children’s social and emotional development [16,17]. Moreover, advice and emotional support from grandmothers on infant feeding were also found to be positively correlated with the grandchildren’s nutritional status [18]. A previous study had shown that conflicted or distant grandmother–parent relationships were associated with more stress for the grandmother [19]; additionally, grandparents’ mental health had also been shown to influence grandchildren’s behavior and health [20]. A study found that better a parent-grandparent relationship was positively linked with adolescents’ life satisfaction [21].

Existing studies have shown that children health statuses were strongly influenced by parents’ health knowledge [22] and parental health genetics [23], such as the occurrence of hypertension [24] and myopia [25]. On one hand, existing studies showed that grandparents’ care could exert a positive effect on the health status of their grandchildren [26,27]. Surveys among Latino students in the U.S. found that students who were cared for by their grandparents were more physical active than those who weren’t [28,29]. On the other hand, several studies also illustrated that living with grandparents was strongly associated with a higher risk of children being overweight and obese [30,31,32,33], and children cared for by their grandparents had higher levels of behavioral and emotional disturbance [34].

Given the above background, no study had simultaneously explored the effects of the living environment, MEFC’s attitude regarding the care of the CMEFC for the GMEFC, and the health statuses of family members on GMEFC’s health statuses, and no study so far had been conducted from the MEFCs’ perspective. Thus, this study aimed to explore the effects of the living environment, health statuses of family members, and MEFC’s attitude regarding the care of their children for their GMEFC on GMEFC’s health statuses in Weifang, China. We hypothesized that there was a positive relationship between the above variables and GMEFC’s health statuses.

## 2. Materials and Methods

### 2.1. Data and Sample

A total of 613 MEFC were included in interviews in Weifang, China, in August 2021. Weifang lies in the east of China and the city achieved a gross domestic product (GDP) of CNY 7010.6 billion in 2021 [35]. In 30 November 2020, the population of children 0–14 years old was 1.63 million, accounting for 17.37% of the city’s total population [36].

Multistage cluster random sampling was employed to select the participants in this cross-sectional study. In the first stage, we selected four districts of the 12 districts and counties as the primary sampling units (PSUs) in Weifang, China. In the second stage, 4 sub-districts were selected in each PSU as secondary sampling units (SSUs). In the third stage, we selected 4 communities in each SSU as final investigation sites. The elderly who were aged 60 years old and migrated to follow their children to Weifang in the selected communities made up the entire sample. The exclusion criteria for participants were: (1) local people; (2) less than 60 years old; (3) unable to communicate.

Face-to-face interviews were conducted by trained university students. A total of 616 samples were interviewed initially; however, 3 participants were excluded due to incorrect or incomplete answers on the questionnaire.

### 2.2. Measurements

#### 2.2.1. Health Status of GMEFC

The health status of GMEFC was assessed by the question of “How is the health of your grandchildren?” on the questionnaire. The original options of the question were divided into: excellent, good, fairly good, average, relatively poor and poor. Taking the distribution of the sample into consideration, the options were converted into “excellent” (the first option) and “not excellent” (the latter five options). Because there may be more than one GMEFC cared by the MEFC in the family, the GMEFC surveyed in this study were babies that the MEFC were currently caring and usually the younger sibling.

#### 2.2.2. Social-Demographic Characteristics

Social-demographic characteristics of GMEFC were collected, including age, sex and whether they were the only child.

#### 2.2.3. Living Environment

Living environment was measured by four indicators, including type of housing, occurrence of elevator malfunction, evaluation of living conditions, and the MEFC’s satisfaction with living conditions.

#### 2.2.4. MEFC’s Attitude about Caring from CMEFC to GMEFC

The MEFC’s attitude about the care of the CMEFC for the GMEFC was measured by two questions. The first question was about the MEFC’s satisfaction with the CMEFC’s time spent caring for the GMEFC, and the second question was whether the MEFC understood and forgave the CMEFC for the limited time spent care-giving. 

#### 2.2.5. Health Status of Family Members

Health statuses of family members were assessed by two questions. The first question was about the health status of the CMEFC, which was measured by asking the participants, “What are the CMEFC’s health conditions?” The second question was about the MEFC’s health status, which was evaluated by asking the subjects, “What is your health status now?”.

### 2.3. Statistical Analysis

Descriptive analysis was used to show the characteristics of the participants. The Chi-square test was employed to clarify the statistical differences of GMEFC’s health status in terms of socio-demographic characteristics, living environment, attitude about care of the CFEMC to the GMEFC and health status of family members, respectively. Statistically significant variables of the Chi-square test were then included in the logistic regression analyses. Four binary logistic regression models with an enter method were adopted to explore the associations between independent variables and health status of the GMEFC. Model 1 included the social demographic characteristics, then the indicators of living environment were brought into Model 2, while MEFC’s attitude about care of the CMEFC for the GMEFC were introduced into Model 3, and finally the variables of health status of family members were added to Model 4. *p*-values of less than 0.05 were regarded as statistically significant. All the statistical analyses above were performed by using SPSS version 24.0 (SPSS Inc., Chicago, IL, USA).

## 3. Results

### 3.1. Basic Characteristics of Participants

A summary of GMEFC’s social-demographic characteristics, living environment, MEFC’s attitude about the care of the CMEFC for the GMEFC, and health status of family members is shown in Table 1. It was illustrated that 74.9% of the GMEFC had excellent health statuses, while 25.1% did not have excellent health statuses. Most GMEFC were male (65.3%), 1 < age < 4 years old (56.1%), and not the only child (63.3%). As for the living environment, 77.3% of the MEFC and GMEFC lived in high buildings, 41.4% had never experienced an elevator malfunction, 61.2% evaluated their living conditions as relatively good and 60.8% were satisfied with their living conditions. With regard to MEFC’s attitude on the care of the CMEFC for the GMEFC, 81.2% felt satisfied, and less than 3% did not understand or forgive CMEFC’s limited time spent care-giving. In terms of the health statuses of family members, 67.9% of the CMEFC had excellent health statuses, and 74.9% of the MEFC did not have excellent health statuses.

The Chi-square test showed that statistically significant differences in the health statuses of GMEFC were found between GMEFC’s sex (*p* < 0.05), GMEFC’s age (*p* < 0.05), whether the GMEFC had siblings (*p* < 0.05), the occurrence of elevator malfunction (*p* < 0.05), evaluation of living conditions (*p* < 0.05), satisfaction with living conditions (*p* < 0.05), MEFC’s satisfaction with CMEFC’s time spent caring for GMEFC (*p* < 0.001), whether MEFC understand and forgive CMEFC’s limited time spent care-giving (*p* < 0.001), the CMEFC‘s health status (*p* < 0.001) and the MEFC’s health status (*p* < 0.001).

### 3.2. Logistic Regression Analysis of the Relationship between the Related Variables and Health Status of GMEFC

Table 2 showed the *p*-values, OR, and 95% CI (CI = confidence intervals) of the association between the statistically significant variables after univariate analysis and the health status of GMEFC, respectively. The collinearity diagnostic results revealed that the tolerances of all independent variables were much greater than 0.1, and the variance inflation factors were far less than 10, suggesting that there was no multicollinearity between the independent variables in the four logistic regression models.

In Table 2, the results of Model 1 presented that the GMEFC’s sex and whether the GMEFC had siblings were statistically significant factors for the health statuses of the GMEFC. When variables of social-demographic characteristics entered into Model 2, these two variables were still statistically significant. Meanwhile, the occurrence of elevator malfunction was also statistically significantly associated with the health status of the GMEFC in Model 2. The results of Model 3 showed that these statistically significant variables in Model 2 were still statistically significant in Model 3; moreover, the MEFC’s satisfaction with the CMEFC’s time spent caring for the GMEFC and whether the MEFC understand and forgive the CMEFC’s limited time spent care-giving were found to be statistically significantly with the health status of the GMEFC. In Model 4, the GMEFC’s sex, whether the GMEFC were the only children, the occurrence of elevator malfunction, the MEFC’s satisfaction with the CMEFC’s time spent caring for the GMEFC, whether the MEFC understand and forgive the CMEFC’s limited time spent care-giving, the health status of the CMEFC, and the health status of the MEFC were statistically significantly associated with the health status of the GMEFC.

Specifically, the GMEFC who were not only children (*p* = 0.006, OR = 1.896), the CMEF who had excellent health statuses (*p* < 0.001, OR = 8.969), and the MEFC with excellent health statuses (*p* = 0.017, OR = 2.232) were more likely to with excellent health statuses. In contrast, the GMEFC who were female (*p* = 0.048, OR = 0.628) or occasionally experienced an elevator malfunction (*p* = 0.048, OR = 0.537), the MEFC who were dissatisfied with CMEFC’s time spent care-giving (*p* = 0.001, OR = 0.365), and the MEFC who did not understand or forgive CMEFC’s limited time on caring (*p* = 0.009, OR = 0.504) were less likely to have GMEFC with excellent health statuses.

## 4. Discussion

### 4.1. Health Status of GMEFC in Weifang, China

A total of 74.9% of the GMEFC (459/613) had health statuses rated as “excellent”, while 25.1% of the GMEFC (154/613) had health statuses rated as “not excellent” in Weifang, China. This result was similar to the health statuses of children in kindergarten in Lanzhou, China [37], and better than the health statuses of pre-school children in Zhongshan, China [38].

### 4.2. Effect of Independent Variables on GMEFC’s Health Status in Weifang, China

#### 4.2.1. Association between Living Environment and GMEFC’s Health Status

This study found that the GMEFC’s health statuses were related to the living environment. In detail, the occurrence of elevator malfunction was found to be associated with the GMEFC’s health status, while their evaluation of living conditions and their satisfaction with living conditions were not. Some previous studies had shown that children’s health statuses were compromised by poor housing [39]. A study among deaf children and children with an intellectual disability in China showed that long corridors and elevators with spaces behind them are not very safe [40]. However, no studies have discussed the relationship between elevator breakdowns and the health statuses of the children from migrant families. The findings of this study suggest that those occasionally experiencing elevator breakdowns were less likely to have excellent health statuses compared to families without elevators, which was similar to a study on older adults [41].

#### 4.2.2. Association between MEFC’s Attitude about Care of the CMEFC for the GMEFC and GMEFC’s Health Status

The result of this study demonstrated that the MEFC’s attitude about care of the CMEFC for the GMEFC was associated with the health status of the GMEFC. Specifically, the MEFC who were dissatisfied with the CMEFC’s time spent care-giving, and the MEFC did not understand and forgive the CMEFC’s limited time spent care-giving were less likely to have GMEFC with excellent health statuses. This showed the influence of family atmosphere on the GMEFC ‘s health statuses; that is, the MEFC’s higher satisfaction and understanding of the CMEFC’s care-giving indicated the more harmonious relationship [15], and less conflict in the mother–grandmother relationship benefited children’s social development directly and indirectly via a reduction in mothers’ negative parenting behaviors [42].

#### 4.2.3. Association between Health Status of Family Members and GMEFC’s Health Status

This study found that the health status of family members was correlated with the GMEFC’s health status. In detail, the CMEFC who had excellent health statuses were more likely to have GMEFC with excellent health statuses, and the MEFC who had excellent health statuses were more likely to have GMEFC with excellent health statuses. Our results above were similar to a study in Kenya which showed that mothers who were overweight or obese had higher odds of having children who were overweight or obese [43], as well as research in Finland that found the children who had newly diagnosed type 1 diabetes were influenced by grandparents with type 2 diabetes [44]. Moreover, a study in the U.S. found that grandmothers who were underweight prior to pregnancy had an increased risk of attention-deficit/hyperactivity disorder among their grandchildren [45]. The results of this study illustrated that social and community networks (including family and wider social circles), as one part of the ‘social determinants of health’, played an important role in determining the quality of the health of a population [46].

#### 4.2.4. Association between Social-Demographic Characteristics and GMEFC’s Health Status

This study showed that the GMEFC who were female were less likely to have excellent health statuses, while the GMEFC who were not the only child were more likely to have excellent health statuses. In a country with no clear preference for sons and daughters, the mortality rate of boys under 5 years old was higher than that of girls of the same age [47], which was different from this study. The difference may due to the idea that boys were generally more active in playing and look stronger than girls, which may make the MEFC feel that the girls have less excellent health statuses. A study in China among the children under 18 years showed that being in a two-child household was better for children’s health [48], which was similar to this study.

### 4.3. Implications

The following recommended measures for community and family members could be considered for the improvement of GMEFC’s health statuses. First, the community should ensure a safe and convenient living environment (such as the use of a functional elevator) to maintain the GMEFC’s health. Secondly, the CMEFC should spend more time and energy on care-giving to reduce the physical and mental pressure of the MEFC; meanwhile, the MEFC should also understand the stress experienced by the CMEFC. Both MEFC and CMEFC should communicate more actively and optimistically to create a good family atmosphere for the GMEFC. Finally, the MEFC and CMEFC should increase their health literacy, practice healthy behaviors and have healthy lifestyles to maintain good health statuses and ensure the health of the GMEFC.

### 4.4. Limitations

This study had several limitations. Firstly, both the health status of the GMEFC and the health status of the CMEFC were obtained and assessed by the MEFC due to the young age of the GMEFC and their absence from home during the questionnaire survey of the CMEFC. This may cause the two variables collected in this study to be better than the actual situation in the cultural context of China and result in bias. Secondly, scales on living environment, health status of family members, and MEFC’s attitude about care of GMEFC were not used in this study. Thirdly, in measuring of the “living environment”, we only paid attention to the outdoor living environment, especially the housing, which may be insufficient. Fourthly, influenced by Chinese culture and social reality, the majority of the respondents chose “excellent”; thus, the transformation of the option of the dependent variable (from six options to two options) needs more consideration. Finally, we used data from a cross-sectional study, so a causal relationship cannot be predicted.

## 5. Conclusions

In summary, to the best of our knowledge, this was the first study to explore the effects of living environment, health status of family members, and the MEFC’s attitude about care-giving on the GMEFC’s health statuses. The results of this study indicated that the better the living environment, the better the health statuses of family members, and the more positive the MEFC’s attitude about care-giving, the better health status of the GMEFC. It is our hope that the results of this study could provide empirical reference for communities and family members on the improvement of GMEFC’s health statuses.

## Figures and Tables

**Table 1:** Descriptive analysis of the health statuses of GMEFC in Weifang, China.

Variable	*n* (%)	Health Status of GMEFC		χ^2^	*p*
		Not Excellent	Excellent		
		*n* (%)	*n* (%)		
Total	613 (100.00)	154 (25.1)	459 (74.9)		
GMEFC’s sex				10.400	0.001
Male	400 (65.3)	84 (21.0)	316 (79.0)		
Female	213 (34.7)	70 (32.9)	143 (67.1)		
GMEFC’s age (years)				6.498	0.039
≤1	56 (9.1)	18 (32.1)	38 (67.9)		
1 < age < 4	344 (56.1)	73 (21.2)	271 (78.8)		
≥4	213 (34.8)	63 (29.6)	150 (70.4)		
GMEFC whether were only child				7.821	0.005
Yes	225 (36.7)	71 (31.6)	154 (68.4)		
No	388 (63.3)	83 (21.4)	305 (78.6)		
Type of housing				0.058	0.810
Low building	139 (22.7)	36 (25.9)	103 (74.1)		
High building	474 (77.3)	118 (24.9)	356 (75.1)		
Occurrence of Elevator malfunction				9.213	0.027
No elevator	194 (31.6)	43 (22.2)	151 (77.8)		
Often	31 (5.1)	6 (19.4)	25 (80.6)		
Occasionally	134 (21.9)	47 (35.1)	87 (64.9)		
Never	254 (41.4)	58 (22.8)	196 (77.2)		
Evaluation of living conditions				16.411	0.001
Poor and relatively poor	21 (3.4)	6 (28.6)	15 (71.4)		
Average	100 (16.3)	35 (35.0)	65 (65.0)		
Relatively good	375 (61.2)	99 (26.4)	276 (73.6)		
Extremely good	117 (19.1)	14 (12.0)	103 (88.0)		
Satisfaction with living conditions				10.957	0.004
Average and below	114 (18.6)	36 (31.6)	78 (68.4)		
Satisfied	373 (60.8)	100 (26.8)	273 (73.2)		
Very Satisfied	126 (20.6)	18 (14.3)	108 (85.7)		
MEFC’s satisfaction with CMEFC’s time spent caring for GMEFC				27.286	<0.001
Satisfied	498 (81.2)	104 (20.9)	394 (79.1)		
Average	88 (14.4)	41 (46.6)	47 (53.4)		
Dissatisfied	27 (4.4)	9 (33.3)	18 (66.7)		
Whether MEFC understand and forgive CMEFC’s limited time spent care-giving				22.308 ^a^	<0.001
Very understand	224 (36.5)	34 (15.2)	190 (84.8)		
Relatively understand	373 (60.9)	112 (30.0)	261 (70.0)		
Not understand	16 (2.6)	8 (50.0)	8 (50.0)		
CMEFC’s health statuses				131.510	<0.001
Not excellent	197 (32.1)	107 (54.3)	90 (45.7)		
Excellent	416 (67.9)	47 (11.3)	369 (88.7)		
MEFC’s health statuses				25.869	<0.001
Not excellent	459 (74.9)	139 (30.3)	320 (69.7)		
Excellent	154 (25.1)	15 (9.7)	139 (90.3)		

Abbreviations: MEFC = migrant elderly following children; CMEFC= children of MEFC; GMEFC = grandchildren of MEFC; ^a^: Fisher’s exact test.

**Table 2:** Binary logistic regression for relationships between related variables and health status of GMEFC.

Variables	Model 1			Model 2			Model 3			Model 4		
	Socio-Demographic Factors of GMEFC			Model 1 + Living Environment			Model 2 + MEFC’s Attitude about Caring			Model 3 + Health Status of Family Members		
	OR	95% CI	*p*	OR	95% CI	*p*	OR	95% CI	*p*	OR	95% CI	*p*
GMEFC’s sex												
Male	1.000			1.000			1.000			1.000		
Female	0.563	0.385–0.823	0.003	0.583	0.395–0.861	0.007	0.595	0.395–0.894	0.013	0.628	0.396–0.996	0.048
GMEFC’s age (years)												
≤1	1.000			1.000			1.000			1.000		
1 < age < 4	1.676	0.897–3.132	0.105	1.550	0.814–2.952	0.182	1.407	0.711–2.786	0.327	2.140	0.971–4.718	0.059
≥4	1.230	0.647–2.339	0.528	1.090	0.558–2.129	0.800	0.961	0.473–1.952	0.912	1.149	0.512–2.581	0.736
Whether GMEFC were only children												
Yes	1.000			1.000			1.000			1.000		
No	1.606	1.101–2.344	0.014	1.571	1.062–2.324	0.024	1.696	1.127–2.551	0.011	1.896	1.197–3.003	0.006
Elevator malfunction situation												
No elevator				1.000			1.000			1.000		
Often				1.308	0.492–3.477	0.591	1.407	0.500–3.960	0.517	0.824	0.262–2.590	0.741
Occasionally				0.537	0.320–0.903	0.019	0.507	0.296–0.871	0.014	0.537	0.291–0.994	0.048
Never				0.977	0.603–1.583	0.926	0.910	0.551–1.504	0.714	0.703	0.403–1.226	0.214
Evaluation of living conditions												
Poor and relatively poor				1.000			1.000			1.000		
Average				0.755	0.254–2.240	0.612	0.824	0.265–2.560	0.738	0.845	0.227–3.142	0.801
Relatively good				1.237	0.366–4.175	0.732	1.024	0.292–3.585	0.971	0.839	0.201–3.505	0.809
Excellent				2.805	0.694–11.343	0.148	2.361	0.564–9.888	0.240	1.370	0.271–6.932	0.703
Satisfaction with living conditions												
Average and below				1.000			1.000			1.000		
Satisfied				0.838	0.402–1.745	0.636	0.943	0.447–1.990	0.877	1.337	0.578–3.090	0.497
Very Satisfied				1.081	0.405–2.883	0.876	1.038	0.379–2.840	0.942	1.078	0.348–3.345	0.896
MEFC’s satisfaction with CMEFC ‘s time spent caring for GMEFC												
Satisfied							1.000			1.000		
Average							0.709	0.260–1.933	0.502	0.502	0.161–1.568	0.235
Dissatisfied							0.367	0.218–0.619	0.000	0.365	0.201–0.661	0.001
Whether the MEFC understand and forgive CMEFC’s limited time spent care-giving												
Very understand							1.000			1.000		
Relatively understand							0.244	0.072–0.828	0.024	0.422	0.104–1.710	0.227
Not understand							0.378	0.239–0.599	0.000	0.504	0.301–0.845	0.009
CMEFC‘s health status												
Not excellent										1.000		
Excellent										8.969	5.570–14.443	0.000
MEFC’s health status												
Not excellent										1.000		
Excellent										2.232	1.155–4.315	0.017

Abbreviations: MEFC = migrant elderly following children; CMEFC= children of MEFC; GMEFC = grandchildren of MEFC; OR = odds ratios; CI = confidence intervals.

## Data Availability

The data presented in this study are available on request from the corresponding author. The data are not publicly available due to privacy or ethical.
